# Stakeholder perspectives on Nigeria’s national sodium reduction program: Lessons for implementation and scale-up

**DOI:** 10.1371/journal.pone.0280226

**Published:** 2023-01-13

**Authors:** Olutobi A. Sanuade, Vanessa Alfa, Xuejun Yin, Hueiming Liu, Adedayo E. Ojo, Gabriel L. Shedul, Dike B. Ojji, Mark D. Huffman, Ikechukwu A. Orji, Rosemary C. B. Okoli, Blessing Akor, Nanna R. Ripiye, Helen Eze, Clementina Ebere Okoro, Linda Van Horn, Priya Tripathi, Tunde M. Ojo, Kathy Trieu, Bruce Neal, Lisa R. Hirschhorn

**Affiliations:** 1 Department of Medical Social Sciences, Northwestern University Feinberg School of Medicine, Chicago, Illinois, United States of America; 2 Department of Population Health Sciences, Spencer Fox Eccles School of Medicine at the University of Utah, Salt Lake City, Utah, United States of America; 3 Cardiovascular Research Unit, University of Abuja and University of Abuja Teaching Hospital, Gwagwalada, Abuja, Nigeria; 4 The George Institute for Global Health, University of New South Wales, Sydney, Australia; 5 Cardiovascular Division and Global Health Center, Washington University in St. Louis, St. Louis, Missouri, United States of America; 6 The University of Nigeria, Nsukka, Nigeria; 7 Department of Preventive Medicine, Northwestern University Feinberg School of Medicine, Chicago, Illinois, United States of America; 8 Stanley Manne Children’s Research Institute, Ann and Robert H. Lurie Children’s Hospital of Chicago, Chicago, Illinois, United States of America; 9 Robert J Havey Institute for Global Health, Northwestern University Feinberg School of Medicine, Chicago, Illinois, United States of America; Federal University of Uberlandia: Universidade Federal de Uberlandia, BRAZIL

## Abstract

**Background:**

To reduce excess dietary sodium consumption, Nigeria’s 2019 National Multi-sectoral Action Plan (NMSAP) for the Prevention and Control of Non-communicable Diseases includes policies based on the World Health Organization SHAKE package. Priority actions and strategies include mandatory sodium limits in processed foods, advertising restrictions, mass-media campaigns, school-based interventions, and improved front-of-package labeling. We conducted a formative qualitative evaluation of stakeholders’ knowledge, and potential barriers as well as effective strategies to implement these NMSAP priority actions.

**Methods:**

From January 2021 to February 2021, key informant interviews (n = 23) and focus group discussions (n = 5) were conducted with regulators, food producers, consumers, food retailers and restaurant managers, academia, and healthcare workers in Nigeria. Building on RE-AIM and the Consolidated Framework for Implementation Research, we conducted directed content qualitative analysis to identify anticipated implementation outcomes, barriers, and facilitators to implementation of the NMSAP sodium reduction priority actions.

**Results:**

Most stakeholders reported high appropriateness of the NMSAP because excess dietary sodium consumption is common in Nigeria and associated with high hypertension prevalence. Participants identified multiple barriers to adoption and acceptability of implementing the priority actions (e.g., poor population knowledge on the impact of excess salt intake on health, potential profit loss, resistance to change in taste) as well as facilitators to implementation (e.g., learning from favorable existing smoking reduction and advertising strategies). Key strategies to strengthen NMSAP implementation included consumer education, mandatory and improved front-of-package labeling, legislative initiatives to establish maximum sodium content limits in foods and ingredients, strengthening regulation and enforcement of food advertising restrictions, and integrating nutrition education into school curriculum.

**Conclusion:**

We found that implementation and scale-up of the Nigeria NMSAP priority actions are feasible and will require several implementation strategies ranging from community-focused education to strengthening current and planned regulation and enforcement, and improvement of front-of-package labeling quality, consistency, and use.

## Introduction

Cardiovascular diseases (CVDs) are the leading cause of death globally, accounting for an estimated 18.6 million lives with over three-quarters of those CVD deaths occurring in low- and middle-income countries [1,2]. Nigeria is experiencing a rapid epidemiological transition away from a pattern of predominantly infectious diseases to increasingly non-communicable diseases, especially cardiovascular diseases [3,4]. The overall age-standardized prevalence of hypertension in Nigeria is 38.1% [5], with estimated monthly household expenditures of 13,575.0 Naira for inpatient care and 5,843.1 Naira for outpatient care [6]. An estimated 100,000 (95% UI: 74,00 to 134,000) deaths from CVD occurred in Nigeria in 2017 [7]. High blood pressure is a major risk factor for CVD, and excess dietary sodium or salt intake is associated with elevated blood pressure and risk of CVD [8].

Evidence shows that processed and ultra-processed foods are important contributors to sodium intake at the population level [9–13]. A multi-pronged population salt reduction strategy has been identified as one of the most cost-effective approaches to reducing the burden of CVD [14]. The World Health Organization (WHO) set the global target to reduce salt intake by 30% by 2025 and provided comprehensive, evidence-based advice about the implementation of salt reduction strategies through the SHAKE package [15] Globally, 96 national salt reduction initiatives have been identified [16] with the potential to reduce the risk of premature (<70 years) mortality from noncommunicable diseases (NCDs), including CVD, by 1/3 by 2030, in accordance with Target 3.4 of the Sustainable Development Goals. The approaches include food reformulation to reduce the salt content of products, consumer education, advertising changes, front-of-package labelling schemes, salt taxation, and interventions in institutional settings such as schools [16,17].

The estimated daily salt intake in Nigeria (5.8g/day) [18] is higher than the WHO daily recommendation level (<5 g/day) [19], but the dietary sources of sodium in community-based, representative samples are not well described but will be collected through the Nigeria Sodium Study. To reduce the increasing burden of CVDs including reducing population-level dietary sodium consumption, the Nigerian government published a National Multi-sectoral Action Plan (NMSAP) for the Prevention and Control of NCDs in 2019, which includes policies based on the SHAKE package [20]. The NMSAP salt reduction components targets four key priority actions: 1) limiting the amount of salt in processed foods, and ingredients; 2) restricting how companies can advertise their food, especially to children, to help improve healthy diet; 3) community mobilization and public health campaigns to change how people learn about food, including limiting marketing of unhealthy food and beverages to children, and; 4) education on nutrition in schools to make sure children and their families understand how to have a healthy diet [21]. These NMSAP salt reduction approaches also include standardized, front-of-package food labeling as a priority intervention to increase the effectiveness of the priority actions to promote healthier diets in Nigeria.

Implementing NMSAP priority actions requires political commitment, program leadership, effective partnerships, community, and manufacturer acceptance as well as adoption, and multipronged action across various sectors [20]. Implementation science provides tools to help policy makers and implementers recognize barriers and facilitators to implementation, as well as potential strategies to address barriers or leverage facilitators in the planning, implementation, and stages [22]. During the planning stage, understanding stakeholders’ knowledge and perceptions on the relevant contextual factors is needed to inform implementation strategies designed to increase the implementation outcomes including acceptability, feasibility, adoption, effectiveness, fidelity, and appropriateness of the national sodium reduction program [23]. The current study aims to examine the dietary salt-related knowledge, attitudes, and behavior among stakeholders and population in Nigeria and perspectives on barriers and facilitators and strategies to support the successful implementation and scale-up of the four NMSAP priority actions, as well as front-of-package food labeling in Nigeria. Results can inform the implementation strategies needed to increase the likelihood of success of the work to reduce excess dietary sodium in Nigeria and inform similar work in the region.

## Methods

### Study design and settings

A formative qualitative study was conducted to explore stakeholders’ perceptions on implementation of the NMSAP priority actions on salt reduction. Guides were developed to explore potential barriers and facilitators with suggestions to address these factors and explore implementation outcomes of the NMSAP priority actions. The NMSAP includes policies based on the SHAKE package aims to achieve at least a 30% relative reduction in mean population intake of dietary salt/sodium by 2025. The national target is to specifically reduce salt/sodium consumption to 3 g/day by 2025. Semi-structured interview guides were developed starting with the Consolidated Framework for Implementation Research (CFIR) [24] and RE-AIM framework [25], and then followed by input from experts in the field of implementation science, cardiovascular health, and nutrition interventions (**S1 Table).** This study was reviewed and approved by the Ethics Committee of the Federal Capital Territory in Nigeria (FHREC/2020/01/89/11-09-20), Northwestern University (STU00213707) and the University of New South Wales (HC200807).

### Study participants

Participants were invited through a purposive sampling of identified key actors at the federal, state, or local level who were knowledgeable or influential regarding dietary intake of the Nigerian population and would be involved in various components of the priority actions. Participants were recruited from two states (Kano and Ogun) in Nigeria and the Federal Capital Territory (FCT), Abuja. These states were chosen to align with states where the Nigerian Federal Ministry of Health (FMoH) and the WHO were already working to pilot the implementation of selected NMSAP priority actions. Data were collected through focus group discussions (FGDs) and in-depth interviews (IDIs) between January 2021 and February 2021. Twenty-three IDIs and 5 FGDs (n = 11) were conducted with health professionals (n = 10), federal, state, and local policymakers (n = 9), community leaders (n = 3), food industry (n = 3), international non-governmental organization (n = 3), food retailers/restaurant owners (n = 3), academia (n = 2), and food and drug regulatory body (1).

### Interview procedures

Written informed consent was obtained from all participants before interviews began. Interviews were conducted either face-to-face at participants’ workplace or online via Zoom by four study team members (3 males, 1 female) trained in qualitative methods and working in non-communicable diseases. Among these team members were a cardiologist, family physician, consultant psychiatrist, and social scientist. Field notes were taken during interviews. One author had prior relationships with some stakeholders before the interviews. Interviews lasted for up to 45 minutes with one conducted in Hausa and the remaining ones in English. All stakeholders participated fully, and transcripts were not returned to them for comments. Further details about participant recruitment, interviews, and data management are reported following the Consolidated criteria for Reporting Qualitative research (COREQ) guidelines (**S2 Table**).

### Data analysis

All interviews were transcribed verbatim, with one IDI in Hausa language translated and transcribed by a professional translator. Data were stored in a secured, password-protected server. We conducted directed content qualitative analysis using a combined deductive and inductive approach. Verbatim transcripts were first summarized using a structured template among authors. Summarized memos were then triangulated by three authors to ensure consistency across all memos. Themes captured in the memos included information on stakeholders’ knowledge about salt and hypertension and their perceptions on population knowledge about sources of salt and risk of associated hypertension, and barriers and facilitators to the four main NMSAP priority actions and front-of-package food labeling, and proposed implementation strategies to address these factors. Perceptions on selected implementation outcomes were also explored. Summaries were then consolidated into matrices by participant type to identify overall knowledge and awareness, implementation outcomes, barriers and facilitators, and suggestion for implementation strategies for the NMSAP priority actions and front-of-package food labeling. The consolidated matrices were then synthesized and reviewed by three authors to map barriers and facilitators to implementation outcomes for each of the priority actions. Some authors reviewed the coherence of the synthesis to provide additional rigor and thick description of the synthesis.

### Results

A total of 34 stakeholders participated in the study (67.6% female) whose mean (SD) age was 39.2 (SD) years (**Table 1).** The largest participant group were health professionals (29.5%), followed by local, state, and federal policymakers (26.5%); most worked in the FCT (94.2%).

**Table 1:** Participants demographic profiles.

Characteristics	FGD (n = 11)	IDI (n = 23)	Total (N = 34)
** **	**n (%)**	**n (%)**	**n (%)**
Age, mean	32.3	46.4	39.2
**Sex**			
Male	5 (45.5)	6 (26.1)	11 (32.4)
Female	6 (54.5)	17 (73.9)	23 (67.6)
**Organization type**			
Academia	0 (0.0)	2 (8.7)	2 (5.9)
Community leaders	0 (0.0)	3 (13.1)	3 (8.8)
Food and drug regulatory body	0 (0.0)	1 (4.3)	1 (2.9)
Food industry	0 (0.0)	3 (13.1)	3 (8.8)
Food retailer/restaurant owners	0 (0.0)	3 (13.1)	3 (8.8)
Health professional	5 (45.4)	5 (21.7)	10 (29.5)
Local, state, and federal policymakers	4 (36.4)	5 (21.7)	9 (26.5)
International nongovernmental organization	2 (18.2)	1 (4.3)	3 (8.8)
**Participant location**			
Federal Capital Territory	11 (100.0)	21 (91.4)	32 (94.2)
Kano	0 (0.0)	1 (4.3)	1 (2.9)
Ogun	0 (0.0)	1 (4.3)	1 (2.9)

IDI: In depth Interview, FGD: Focus group Discussion.

### Knowledge about salt and its impact on health

Stakeholders were highly knowledgeable regarding the risks of excess salt intake and recognized this as a problem in Nigeria. They also acknowledged that unhealthy diet population-wide has contributed to the increased prevalence of hypertension and NCDs in Nigeria. However, stakeholders also reported a low level of awareness on the dangers of excess salt consumption among the general Nigerian population. Reported reasons for excess salt consumption in Nigeria included: 1) lack of knowledge of risks associated with excessive amount of salt in food; 2) customs and cultural tastes for high-salt diets; 3) salt as an inexpensive flavor enhancer prompting liberal use in food preparation; 4) urbanization, globalization, and changes in work dynamics associated with reduced consumption of home-cooked meals and increased consumption of commercially prepared cooked foods, including ultra-processed foods.

### NMSAP priority action 1: Limit the amount of salt in processed foods, and ingredients

#### Potential barriers and facilitators

**Table 2** shows the identified potential barriers and facilitators to implementing the first NMSAP priority action, including factors affecting both consumers as well as suppliers and producers of processed foods. Low general awareness on the harmful effects of excess dietary salt intake was noted by many respondents as a barrier to acceptability and perceived appropriateness of NMSAP priority action 1. Respondents mentioned that there was a lack of general knowledge of the level of salt in many commonly consumed foods and seasonings, which was further complicated by lack of knowledge of the recommended daily sodium intake. They also noted that salt was an inexpensive flavor enhancer, thereby making high-salt diets and seasonings more popular, affordable and widely available. Salt’s low cost and corresponding unhealthy diets would further reduce acceptability and adoption of implementing this NMSAP priority action if it resulted in a change in taste or increased cost as mentioned below:

“*Salt is very cheap in Nigeria*, *so it’s something everywhere*. *So, we intend to consume and abuse it a lot*. *When we want to boil our meat to season it, we use salt because we don’t have money for expensive spices. So, we use salt.” [FGD, health professionals]*

**Table 2:** Barriers, facilitators and potential affected implementation outcomes for NMSAP priority action 1.

Barriers (-)/facilitators (+)			Implementation outcomes and effectiveness
Organizing theme	Basic theme	Quotes	
Lack of knowledge on level and impact of salt intake (-)	Low awareness on the harmful effects of excess salt intake on CVD (CL, LSF, AC)	*I believe there’s not much sensitization on salt consumption can increase the risk of chronic disease*. *So*, *there is a need for educating people right from the grassroots like by starting early because some people might just think that at the end of the day*, *it’s when you’re old that the effects will now go to your heart*.*[(IDI*, *local government representative])*.	Acceptability Appropriateness
	Lack of knowledge on the recommended maximum daily sodium intake (CL, AC)	*Most of the time*, *you will find out most people don’t have that knowledge about which one is normal*.* [IDI*, *community leader]* *…*.*but even if they know that salt is not too good for the body*, *but at what quantity*?*[IDI*, *Academia]*	
	Lack of population knowledge on the existence of salt in some foods (e.g., noodles) and seasonings (e.g., bouillon cubes) (CL, RB, AC)	*Most people will tell you that they don’t eat salt*, *but they use Maggi*. *And what is Maggi*? *Salt*. *[IDI*, *community leader]*	
Lower cost of salt and (un)healthy diets (-)	Salt is cheap and therefore used more than other spices which are more expensive (HP)	*Salt is very cheap in Nigeria*, *so it’s something everywhere*. *So*, *we intend to consume and abuse it a lot*. *When we want to boil our meat to season it*, *we use salt because we don’t have money for expensive spices*. *So*, *we use salt*. *[FGD*, *health professionals]*	Acceptability Adoption
	High cost of healthy diets and affordability of high-salt diets because salt is an inexpensive flavor enhancer (HP, CL)	*For the lower economic class*, *a lot of them also find it difficult to purchase fruits and vegetables*, *and you find that they are tilting towards most starch*, *heavy foods*, *because that’s what is easily available*, *and that’s what their finances can procure*. *[FGD*, *local/state/federal government representatives]*	
	Availability and affordability of high-salt diets because salt is an inexpensive flavor enhancer (HP, CL)	*Because there are fruits and vegetables that are very low in sodium*, *but they are very*, *very expensive*. *And*, *when you look at the other way*, *like meat*, *the red meat is a little bit cheaper and that is why a lot of people eat a lot of red meat*. *[IDI*, *health professionals]*	
Mistrust in government due to poor implementation of existing food and dietary policies (-)	Lack of trust in the implementation of food and dietary policies based on government’s lack of implementation of previous or existing policies (CL, HP, DT)	*So*, *you know*, *sorry for saying this*, *sometimes*, *in our country*, *when they (the government) say they will do something*, *it will be taken seriously at first*, *but later there will be lapses*. *We don’t know whether it is from those in charge of the policy*, *or wherever it comes from*, *only God knows*. *The reason I said this is*, *for instance this issue of [unintelligible]*, *they said they will feed our children for free*. *They started it*.* *.* *.*but it is no longer being carried out*, *it has stopped*. *Our problem is just that if they want to do something*, *if the government said it is introducing something*, *they should make every effort to see it to the end*. *But they will start and then off it goes*. *[IDI*, *community leader]*	Acceptability Effectiveness Fidelity
Concern about loss of customers because of changing taste (-)	Possibility of resistance from food industry due to possible reduction in profits/sales which may arise due to concerns that reduced salt content in food may affect consumers’ taste of the food and subsequently have an impact on sales/profit (INGO, FR)	*The challenge that we would have as restaurant owner is that your taste is something that people are already familiar with*. *And*, *there’s a particular way that they expect it to taste*, *if it doesn’t taste that way then you are bound to be losing some costumers and all of that*.*” [IDI*, *food retailer/restaurant owner]*	Adoption Feasibility
Higher cost of low-sodium foods to consumers (-)	Possibility of increase in cost of low-sodium foods when the policy on limiting the amount of salt in foods is implemented	*It will be difficult for people to accept this policy if it is going to involve Nigerians spending their resources on it*. *And looking that we are finding life a little hard financially… So*, *if this policy is not going to involve Nigerians spending money before they can afford to buy foods with low sodium*, *then it is going to be very successful; otherwise*, *it will not be widely acceptable*, *they (Nigerians) will begin to frustrate and call you names*. *[IDI*, *academia]*.	Acceptability Adoption Reach
Lack of salt replacement (-)	Non-availability of salt substitutes that can be presented to consumers (HP)	*If you tell people to stop taking excess salt*, *then you need to give them an alternative*, *definitely*. *Rather than buy*, *probably*, *canned food*, *processed food*, *do we have an alternative that can deliver on the kind*, *the same kind of taste*, *that they are looking for*, *or what they are looking*? *No*! *[IDI*, *health professional]*	Feasibility Acceptability
Cultural and social practices (-)	People are accustomed to foods and preparation methods which supports excess salt intake (CL, FI, LSF, INGO, HP)	*If we consider that aspect*, *particularly those in the rural areas*, *there is no way you can separate them from such unhealthy food*, *they don’t even know the dangers of eating salt up till now*. *For those of us in the city*, *there are a few of us that if you tell them about the dangers of salt*, *they will say*, *“Please*! *Forget all those talks*. *Didn’t we meet our parents eating the same foods*?*” [IDI*, *community leader]* *There are several diets that link to our culture that are not healthy*. *[IDI*, *health professional]*	Acceptability Feasibility Fidelity
	Existence of cultural practices that associate excess salt intake with taste and meal enjoyment/satisfaction (CL, HP)	*‘Most people will tell you that they don’t eat salt*, *but they use Maggi*. *And what is Maggi*? *Salt*. *[IDI*, *health professional]* *The part that will be hardest to change is the attitude*. *Because already we have an attitude that ahh*, *I want this food to be tasty*, *some will say*, *“Ah*, *if I don’t put taste*, *I will not enjoy the meal*. *[IDI*, *health professional]*	
Existence of multiple sources of salt (-)	Exposure to multiple sources of salt in foods (e.g., home-cooked foods, salt added at the table, salt use as preservatives, sea foods, processed foods, restaurants, salt-based seasonings, fast foods) which people may not recognize as high sodium thus making it difficult for people to accurately identify what the main sources of salt in their diets are (LSF)	*You see our people*, *when they want to preserve meat*, *they will put a lot of salt*. *Yeah*, *we know*, *they even taught us in school then that one of the ways of preserving food is by salting so that flies or bacteria will not develop on it*. *This same people that are putting salt in meat*, *preserving salt in meat*, *and there’s also this Yoruba locust beans*, *there is this particular one that they preserve with salt*. *This same people will want to cook soup*, *they will add salt*, *they want to cook rice*, *they will add salt*, *forgetting the cumulative effects of the salt over time*. *[IDI*, *federal government representative]*	Acceptability Effectiveness Feasibility
Non-availability of standard salt measurement in home-cooked meals (-)	Difficulty in measuring the amount of salt in home-cooked meals (HP)	*But aside that*, *people do not know whether or not salt is good for the body*. *But even if they know that salt is not too good for the body*, *but at what quantity*? *[IDI*, *health professional]*	Acceptability Feasibility Fidelity
	Lack of existence of general standard measure of salt for home cooked meals and which promotes discretionary salt use (i.e. people adding salt based on their taste buds) (HP)	*You see some people after preparing their food*, *they will put a bowl or a bottle of salt by the side*, *and they will now start adding another salt*, *added salt to it*. *That’s one*. *Also*, *when they want to preserve meat*, *they will put a lot of salt*. *Yeah*, *we know*, *they even taught us in school then that one of the ways of preserving food is by salting so that flies or bacteria will not develop on it*. *These same people that are putting salt in meat*, *preserving salt in meat*, *and there’s also this Yoruba locust beans*, *there is this particular one that they preserve with salt*. *These same people will want to cook soup*, *they will add salt*, *they want to cook rice*, *they will add salt*, *forgetting the cumulative effects of the salt over time*. *[IDI*, *federal government representative]*	
Lack of knowledge and poor design of nutrition labels (-)	Low interest/practice of looking at nutrition labels (INGO, FR, AC, LSF).	*(W)e only consume what we want to consume*, *without taking care to know the substance that that particular food is made up*. *You can walk in to either a supermarket and buy a sardine*, *you consume the sardine without checking what is written on the sardine*, *the quantity of salt in it*, *the quantity of this and that*. *[IDI*, *food industry]*	Acceptability Effectiveness Feasibility
	Poor design of nutrition labels (Tiny lettering of nutrition facts labels making it difficult to read (INGO, HP)	*Nigerians hardly look at food labels but rather depends on what they hear verbally*. *The labels also have tiny readings therefore discouraging readers [IDI*, *health professional]* *For one*, *the labels are very small*, *they take a very small proportion of those packs*. *So*, *most times*, *it’s even difficult to see what is written there*. *[FGD*, *international non-governmental organizations]*	
	Limited understanding of information on nutrition labels	*They look at all the things that are there but most of them don’t even look at it and see whether they have large amount of salt in the diet or not”*. *What most Nigerians check is the expiry dates*. *How many Nigerians even know what the normal amount of salt is*? *[IDI*, *local government representative]*	
	Misleading and deceptive labeling (FI)	*They (consumers) just feel that these people just try to sell their market by putting in what suit them*. *For example we buy bread and you see a lot of bread contains a label “no saccharin or no sugar added or no saccharin added or no bromate added”; but then*, *that particular food contains bromate*.* *.* *. *[IDI*, *local government representative]*	
	Use of technical terms- labels are too complex to understand due to use of technical terms (FI)		
	Absence of nutrition facts label in processed foods (HP, FI)	*A large number of these food are not even labeled neither are the contents or ingredients outlined*. *For example*, *when somebody takes eggroll*, *meat pie and things like that*, *the content and component are not mentioned*. *We just eat virtually what we find on the street so it’s a problem*. *[IDI*, *health professional]*	
	Lack of trust in nutrition labels because labels may not be a true reflection of the food content (FI, LSF, AC, D, INGO)	*Yes*, *but not all labels*. *Most especially the locally made because they feel from experience like some people have said*, *they feel that sometimes even if the thing expire*, *they remove the label and put a new one*. *So*, *some people don’t trust it*. *[IDI*, *local government representative]* *Although people trust some labels*. *However*, *people do not trust all the food labels*, *especially the locally made products*, *because they think that sometimes even if the product expires*, *they (food manufacturers) can remove the label and put a new one*. *So*, *some people don’t trust it*. *[IDI*, *local government representative]*	
Perceived long-term implementation of salt reduction program (-)	Perceived long duration of implementing salt reduction strategies in processed/home-cooked meals due to the long time it takes to translate evidence into policy and the longstanding food traditions and tastes (HP)	*Well*, *one of the difficult aspects to change in respect to the meal would be*, *let me use the word …*, *some of the processed food and especially the ingredients*. *In a situation whereby a large number of people feel that things like salt*, *seasoning must be in every food*, *and their high content is a sign that such food is tasty*. *So*, *it would take a long time or very difficult for many people to change from such*. *[IDI*, *health professional]*	Adoption Feasibility Effectiveness

CL- Community leaders; FI- Food industry; LSF- Local, state, and federal government; INGO- International NGOs; FR- Food retailers; HP- Health professionals; AC- Academia, RB- Regulatory bodies, DT- Dietician.

Mistrust in government-led initiatives also emerged as a major barrier which may impact acceptability and effectiveness of NMSAP priority action 1. Participants noted that the Nigerian population may find it difficult to trust any policy developed by the government targeted at salt reduction because of implementation failures of previous and existing food and dietary policies.

…*(I)n our country*, *when they (the government) say they will do something*, *it will be taken seriously at first*, *but later there will be lapses*. *We don’t know whether it is from those in charge of the policy*, *or wherever it comes from*, *only God knows…Our problem is just that if they want to do something*, *if the government said it is introducing something*, *they should make every effort to see it to the end*. *But they will start and then off it goes*. *(IDI*, *community leader)*

Participants noted potential barriers from food manufacturers and store and restaurant owners, which could reduce adoption. One barrier included potential loss of consumers due to increased cost or change in taste. For instance, some participants mentioned that bread and packaged noodles are important staple foods in Nigeria that have contributed to excess salt consumption. Reducing salt in these foods was perceived to change flavor or reduce consumer consumption, which may threaten supply-side profits from these products.

*The challenge that we would have as restaurant owner is that your taste is something that people are already familiar with*. *And there’s a particular way that they expect it to taste*. *If it doesn’t taste that way*, *then you are bound to be losing some customers and all of that*. *(IDI*, *food retailer/restaurant owner)*

Other potential barriers identified by participants which may affect acceptability, adoption, perceived appropriateness, and effectiveness of this priority action included: lack of knowledge on and poor design of nutrition facts labels; reducing consumers ability to choose food based on salt content; lack of availability of low-sodium or other salt substitutes as flavor enhancers (although some also reported that the culturally-grounded use of spices in some part of the country could reduce this challenge); cultural and social practices to foods and preparation methods which includes excess salt use and intake; existence of multiple sources of salt (e.g., home-cooked meals, processed foods, meals from restaurants, salt-based seasonings) which would make the reduction of total intake complex; and non-availability of standard salt measurement in home-cooked meals including those where high-salt seasonings are used.

#### Strategies to implementing NMSAP priority action 1 (S3 Table)

Participants highlighted strategies which could help overcome some barriers. To address lack of knowledge around excessive salt intake and guidance, participants suggested generating evidence on the amount and sources of salt being consumed by Nigerians. These data would be useful to justify this and other salt reduction strategies and could be used for consumer education. Secondly, participants believed that this NMSAP priority action may be more acceptable to overcome the distrust of national-led initiatives by involving healthcare providers as more trusted information sources for dissemination and to informing patients and the general populace on the dangers of unhealthy, high-salt diets.

Key informants identified several other strategies to address the potential barriers to implementing NSMAP priority action 1, many of which are already included in the NMSAP overall document (Nigeria Federal Ministry of Health 2019). Participants suggested developing and implementing policies that mandate sodium limits across the food industry. This approach would minimize concerns unfairly targeting specific manufacturers because the policy would be broadly applicable and thus would create a level playing field. Participants also suggested that poor implementation of existing food and dietary policies could be tackled through the development of strong government leadership, regulatory processes, and accountability mechanisms. Lack of knowledge on and poor design of nutrition facts labels could be addressed if the government implemented mandatory nutrition labeling standards to improve content and format (i.e., compulsory declaration of food ingredients, components, and relative healthfulness), including consumer-friendly front-of-package labeling. Reflecting on priority action 3, community mobilization and public education campaigns on the dangers of excess salt intake were identified by many participants to further address lack of population knowledge on the level and the impact of excess salt intake.

### NMSAP priority action 2: Change how companies advertise their food, especially to children, to help improve healthy diets

#### Potential barriers and facilitators

Six major factors were identified by participants as potential barriers or facilitators, to the implementation of the second NMSAP priority action of changing how companies advertise their foods, especially to children (**Table 3**). According to the participants, weak advertising regulations in Nigeria made it difficult to control what was advertised on different media platforms, reducing potential adoption, fidelity, and effectiveness of this priority action. Similarly, there was a perception among respondents that not using appropriate communication channels to drive this priority action could potentially impact adoption, fidelity, and effectiveness. Lack of consumer trust in advertising regarding the content of the products being advertised was also noted as a barrier to effectiveness.

*There are some advertisements about bread that shows that it is sugar-free and bromate-free*, *but is it true that what is truly contained in it is exactly what they are advertising*? *(IDI*, *academia)*

**Table 3:** Barriers, facilitators and potential affected implementation outcomes for NMSAP priority action 2.

Barriers (-)/facilitators (+)			Implementation outcomes and effectiveness
Organizing theme	Basic theme	Quotes	
Poor advertising regulations (-)	Poor advertising regulations in Nigeria making it difficult to control what is being advertised on different media platforms (LSF, INGO, CL, Dieticians)	*‘I think we don’t have regulation in advertisement in Nigeria*. *I’ve noticed that*. *Sorry*, *I’m saying this but I…*, *and we have a very big gap in the way we advertise*. *(IDI*, *health professional)* *Yes*. *You know*, *like those fast noodles*, *you can see now*. *So*, *like this indomie*, *coke*, *they sponsor football clubs*, *they will sponsor children quiz; school will call them during their interhouse*, *they will pay the school*, *and then they will put their stand*. *So*, *such things should be regulated; they should not be allowed to do so; so*, *children will now know*. *And then*, *television stations should be told not to advertise*. *There should be a policy to curtail their adverts*, *unless they will change the dietary content of that food*. *(IDI*, *health professional)*.	Adoption Fidelity Feasibility
Poor communication on the need for this NMSAP strategy (-)	Poor communication (by policy makers/experts) on the need to change how food companies advertise their food, especially to children, thus making it easy for this strategy to be hijacked by people with different vested interest in salt reduction (HP)	*The way it’s being communicated*. *It’s very easy for information to be deformed or to go*, *for people who have a way of changing information*, *because they want to achieve certain things*. *We should not allow people hijack this*, *and one of the ways to do*, *one of the things to do*, *is to ensure that the experts are allowed to always speak to this issue*.* [IDI*, *health professional]*	Adoption Fidelity Effectiveness
Consumer distrusts in brands and advertising (-)	Lack of consumer trust in advertising as regards the content of the products being advertised (PAG)	*There are some advertisements about bread that shows that it is sugar free and bromate free*, *but is it true that what is truly contained in it is exactly what they are advertising*? *[IDI*, *academia]*	Acceptability (trust) Effectiveness Fidelity
Favorable existing advertising strategy on smoking (+)	Tobacco products advertising approach has led to reduction in smoking. This creates the right environment for similar approach to be used for salt reduction. (LSF, INGO)	*What will make this acceptable to me is that if*, *if I am properly educated that this thing is detrimental to my health*, *and I believe many Nigerians also once they know that this thing is detrimental to their health that would help*. *And also*, *if the*, *if the company can also specifically mention and say okay this particular food contains so*, *so amount of salt*, *which may be detrimental to your health*, *at least that will actually raise a red flag for people that are taking it*. *Like what they do in tobacco*. *Yes*. *they must*, *they say they must advertise tobacco*, *but you must give that caution or warning that this thing is harmful to your health*. *(IDI*, *academia)*	Adoption Effectiveness
Possible resistance from food industry due to perceived reduction in sales (-)	Possible resistance from the food industry due to the perceived threat on their sales and consequently their profits. (-) (LSF, INGO), HP		Adoption Feasibility
	Lack of responsiveness from the food industry making it difficult to limit the amount of salt in processed foods (HP)		Adoption
High media presence in Nigeria/ collaborations with advertising companies (+)	There is a high media presence in Nigeria. Hence, collaboration with advertising companies to promote the need for salt reduction will be an effective strategy towards salt reduction because of the wider reach of these advertising companies (HP, INGO)	*People look at this advertising companies so much–on news*, *radios*, *whatever; they give out handouts*, *they advertise with products*, *they give samples*, *you understand*, *free things*, *scholarships*. *So*, *they can make them friends*, *to do the right thing*, *and give them space to advertise and show what changed; the public will listen to them*. *(IDI*, *health professional)*	Adoption Effectiveness Feasibility

CL- Community leaders; FI- Food industry; LSF- Local, state and federal government; INGO- International NGOs; FR- Food retailers; HP- Health professionals; AC- Academia, RB- Regulator bodies, DT- Dietician.

Possible resistance from the food industry due to perceived threats on sales and consequently profits was also identified by participants as a potential barrier that could affect adoption. In contrast, one potential facilitators of implementing this NMSAP priority action is the existence of a favorable and effective advertising strategy to reduce smoking. This experience has created a favorable environment for similar advertising regulations related to excess dietary salt reduction in Nigeria. Moreover, participants stated that there was a large media presence in Nigeria which could facilitate adoption and effectiveness of this NMSAP priority action. Widespread media presence would facilitate rapid implementation and reach of modified advertisements, resulting in better effectiveness.

### Strategies to implementing the NMSAP priority action 2 (S4 Table)

To address limited advertising regulations in Nigeria, participants suggested that the Nigerian national government develop policy regulations on how the food industry advertises its products, including restricting where and when advertising can be done, how much, and how prominent the advertising should be. Also, participants noted that advertising agencies can strengthen advertising regulations by ensuring that the food industry conforms to laws and regulations that govern food advertisements. Similar to other priority actions, respondents identified the need to improve and mandate front-of-package nutrition labeling to help consumers understand what is in the products to increase demand. Other strategies focused on enforcement including sanctions of companies that do not conform to advertising regulations, as well as monitoring and surveillance of food advertisements. Participants mentioned that using a similar approach to tobacco advertising regulations could be an effective strategy to change how companies advertise their foods, especially to children.

*What tobacco has done is to enact a law that prohibits advertisement to children and anywhere around where children are prone to be available… I think the same can apply to food and beverage industries that are quite high or rich in salt and trans-fat*, *the same can apply to them as well*. *(FGD*, *local/state/federal government representatives)*

According to participants, strategies were also needed to increase the effectiveness of strengthening advertising regulations on food through strategies to strengthen the communication channels. In addition to priority action 3 (public health campaigns) these included: 1) use of organizations drawn from local community councils to educate and sensitize the public before policy implementation to increase acceptability and effectiveness; and ensuring that advertisements included information about the danger of excess salt in advertised foods. In addition, participants suggested educating the food industry on the need for salt reduction, increasing government advertising support for the food industry (e.g., through subsidies of government owned media houses) to incentivize change, and involving the food industry in government plans to change advertising approach on salt reduction as strategies to address possible resistance to adoption from the food industry in implementing the second NMSAP priority action.

### NMSAP priority action 3: Public health campaigns to educate people about healthy foods, including those low in salt

#### Potential barriers and facilitators

Poor internet services, high cost of public health campaigns, high level of illiteracy in Nigeria, and cultural practices which support excess salt consumption were identified as potential barriers that could affect effectiveness, feasibility, acceptability, and perceived appropriateness of the third NSMAP priority action (**Table 4).** Additional barriers included public lack of awareness of excess salt risks, ideal salt intake levels, poor food labeling, and distrust of some government initiatives. Participants noted some facilitators including public interest in health issues in Nigeria, thus providing a good platform for public campaigns to educate people about foods.

*If there is any information on health*, *our people do listen to them*. *They welcome them*, *they will sit and then they will talk to them*, *and they will hear*. *(IDI*, *community leader)*

**Table 4:** Barriers, facilitators and potential affected implementation outcomes for NMSAP priority action 3.

Barriers (-)/facilitators (+)			Implementation outcomes and effectiveness
Organizing theme	Basic theme	Quotes	
Poor internet services (-)	Internet services in rural areas are poor and expensive (CL)	*Well*, *internet is one way*, *people use it a lot*, *and it is helpful*, *except for those in the rural areas*, *sometimes they tend to have very poor services*. *(IDI*, *community leader)*	Effectiveness Feasibility
Public interest in health issues (+)	Nigerians like to be informed about health issues (CL, FI, INGO)	*If there is any information*, *our people do listen to them*. *They welcome them*, *they will sit and then they will talk to them*, *and they will hear*. *(IDI*, *community leader) *	Acceptability Effectiveness Appropriateness
High cost of public health campaigns (-)	Implementation of this strategy requires a lot of funding (INGO, LSF)	*Yeah*, *you know*, *public awareness is very crucial in every public health intervention that we’re*, *that you know*, *might want to implement; but it is so*, *unfortunately it is even the least that is being implemented to be honest*, *because*, *it’s every expensive*. *If you want to put it out there say from the regular media outlet*, *the cost is prohibitive and nobody even wants to go there*. *So*, *we have to be very intentional and strategic in the way we promote or advocate for public awareness*. *(IDI*, *local government representative)* *So*, *I think this is probably the easiest to implement among all the four strategies*, *the public health campaigns*. *The only challenge will be the funding required to push it and expand across the country*. *[FGD*, *international non-governmental organizations]*	Feasibility
There are existing government institutions tasked with public health campaigns (+)	There are existing structures for public health campaign (INGO)	*There are already*, *there are several structures*, *the health promotion departments*. *If you go down to the state level*, *we have the state health educators*. *So*, *I think the structure is already well grounded*. *[FGD*, *international non-governmental organizations]*	Effectiveness
	Existence of National Orientation Agency (LSF)	*Even though government has the avenue*, *you know like the National Orientation Agency*, *but they are not being used to the maximum*. *Because this is an organization that has presence in every local government*, *every ward supposedly*, *so whatever message you want to pass ordinarily we should be able to get every message to be passed to everybody in this country through the National Orientation Agency*. *But my experience is that*, *it has not really happened*, *I don’t know whether it is because they are not properly engaged or whether they also don’t have the true capacity to do their statutory responsibility*. *(IDI 014)*	
High level of illiteracy in Nigeria (-)	High level of illiteracy in Nigeria (FR, LSF)	*So*, *it depends on who is leading*. *It will take a long time*. *It’s not something you do*, *because of illiteracy in Nigeria*, *it is not something you do for six months or one year; it should be part and parcel of the TV media*, *the radio media*, *in different languages and what not*, *for years because of illiteracy*. *(IDI*, *federal government representative)*	Acceptability Appropriateness
Culture (-)	Cultural background (HP)	*For now*, *we may not see anything exactly that will make it unacceptable except for the issue of maybe*, *culture and*, *err*, *mainly cultural background*, *where people may have certain things to do with salt*, *which people believe that is a necessity*. *But outside that*, *I don’t see it being any hindrance to anybody*. *(IDI*, *health professional)*	Appropriateness Acceptability

CL- Community leaders; FI- Food industry; LSF- Local, state and federal government; INGO- International NGOs; FR- Food retailers; HP- Health professionals; AC- Academia, RB- Regulator bodies, DT- Dietician.

Further, participants highlighted that use of existing government institutions and structures, such as the National Orientation Agency that is tasked with the responsibility of mass awareness campaigns including public health campaigns, to facilitate rapid adoption and effectiveness of this NMSAP priority action.

### Strategies to implementing the NMSAP priority action 3 (S5 Table)

Many participants mentioned that to implement NMSAP priority action 3, public health campaigns should be conducted in local languages and carried out in platforms where different audience could be reached such as traditional media (e.g., TV, radio), social media, religious institutions (e.g., churches, mosques), and public spaces (e.g., markets, motor parks). Participants mentioned that targets of public health campaigns should include both children and parents, as well as religious leaders, traditional rulers, opinion leaders, and ethnic leaders.

*What I would just want to add to that is that when we are going*, *when we go below the industry, the stakeholders, the whatever, when we are getting to the lower cadre*, *we should try to speak to them in their own languages*…*Let it get to the languages that everybody understands*. *And then, the message will be passed across*. *(FGD, food regulators)*

Participants highlighted the need for multi-sectoral collaboration and involvement to promote public health campaigns for implementing this NSMAP priority action. These stakeholders should include local, state, and federal government, local and international NGOs, and government agencies, such as the National Orientation Agency, Ministry of Women Affairs, and Ministry of Education. Use of trusted sources such as religious leaders, ethnic leaders, community health workers, community mobilizers, health professionals, community health volunteers, teachers, and social networks was identified as a key strategy to increase acceptability and effectiveness. Other strategies included: grassroot mobilization to change how people learn about food; use of other influencers such as music and movie stars, celebrities, and politicians to champion salt reduction in public health campaigns; and framing of public health campaign messages in a way that shows the impact of excess salt intake on health (e.g., high blood pressure, cardiovascular disease) and enlightens consumers on how to limit daily salt intake.

### NMSAP priority action 4: School-based nutrition education to make sure children understand how to eat a healthy diet

#### Potential barriers and facilitators (Table 5)

This priority action was highlighted as the most important and perhaps most likely to be effective according to respondents. Barriers highlighted by participants included bureaucracy by school authorities and teachers’ lack of knowledge on appropriate salt intake levels, which could affect adoption and feasibility. Conversely, participants stated that children influence their families, which could be leveraged on to facilitate effectiveness of this NMSAP priority action. They emphasized that integration of nutrition education into the school curriculum may have a long-term impact because children generally trust their teachers and believe that what is taught in school is true and could favorably influence the dietary behaviors of their families. Some participants mentioned that there were existing government school feeding and health programs underway in some schools in Nigeria. The successful adoption of such programs could influence acceptance, feasibility, and effectiveness of this NMSAP priority action more broadly.

**Table 5:** Barriers, facilitators and potential affected implementation outcomes for NMSAP priority action 4.

Barriers (-)/facilitators (+)			Implementation outcomes and effectiveness
Organizing theme	Basic theme	Quotes	
Bureaucracy by school authorities (-)	Bureaucracy by school authorities (CL)	*(I)t’s bureaucracy by the school authority*. *You know*, *before you could enter a school*, *you must pass through school authority that will allow you to*, *not necessarily individual schools*, *I mean the central governing body of school*, *let me use Local Education Authority or FCT Education Secretariat*, *who will now give that order to move in*. *I think that bureaucracy would be a problem*.* [IDI*, *community leader]* *(W)e cannot introduce anything in the school without involving*, *could be the owner of the school*. *[IDI*, *community leader] *	Feasibility Adoption
Children have a lot of influence on their families (+)	Children have a lot of influence on their families (HP)	*Because*, *I tell you*, *the children have a lot*, *subtle way of influencing decisions in the family*. *So*, *if they are*, *they know the importance of salt*, *you can imagine if it is now your child that is coming home as an adult to give you information about basic things that you should know*, *you will feel very ashamed*, *and then you’re forced to comply*. *[IDI*, *health professional]*	Effectiveness
Lack of knowledge on appropriate salt intake levels (-)	Some teachers are not aware of appropriate salt intake levels (-)	-	Adoption
Existence of school feeding program and school health programs in some schools (+)	There are existing government school feeding program in some schools, as well as school health programs run by adolescents and young people (INGO)	-	Adoption Feasibility Effectiveness

CL- Community leaders; FI- Food industry; LSF- Local, state and federal government; INGO- International NGOs; FR- Food retailers; HP- Health professionals; AC- Academia, RB- Regulator bodies, DT- Dietician.

### Strategies to implementing the NMSAP priority action 4 (S6 Table)

Participants reinforced the need to integrate nutrition education within the school curriculum as a major strategy to ensure feasibility and adoption. Integrating nutrition education into school curriculum would achieve a sustained and prolonged healthful influence on children and increase the likelihood that low salt intake is maintained into adulthood. Participants cautioned that such a nutrition education program should be simple, and easy to understand by children and applied whenever and wherever school food is served.

To address the bureaucracy by school authorities, participants suggested the need to work with teachers and the school administrators and to engage the Ministry of Education for the institutionalization of school-based activities and policies on salt reduction in Nigeria. Further, training of chefs in proper nutrition on the need to prepare low-salt meals and training of teachers to be able to teach healthy diets to school-children are key strategies to improving knowledge on salt intake levels among school staff. Since there are existing school nutrition and health programs, these could be leveraged to facilitate the provision of nutritious diets that are low-salt diets, but flavor enhanced to children. Other strategies mentioned by participants included teaching children on the benefits of healthy eating, culinary arts, and health risks associated with poor diet quality and excess salt intake. These messages could be conveyed using social media, poems, debate competition, cartoons, radio jingles, and use of ambassadors and celebrities and during classroom sessions, practical demonstrations of healthy and low-salt diets in schools.

### Food labeling

#### Potential barriers and facilitators (S7 Table)

Food labeling is included in the NMSAP goal 2, which focuses on promoting healthy diet in Nigeria, and relates to NMSAP priority actions 1–3. Participants noted potential barriers to strengthening food labeling in Nigeria included: absence of nutrition facts label in processed foods; tiny lettering of nutrition facts labels; misleading and deceptive labeling; difficulty of understanding nutrition labeling due to use of technical terms; lack of trust in food labels because labels may not be an accurate reflection of the food content; lack of knowledge on the benefits of reading food labels; and checking food products for other information, such as expiry dates, calories, sugar levels, and Nigerian Agency for Food and Drug Administration and Control code rather than information on salt.

*They (Nigerian community members) look at all the things that are there but most of them don’t even look at it and see whether they have a large amount of salt in the diet or not*. *What most Nigerians check is the expiry dates*. *How many Nigerians even know what the normal amount of salt is*? *(IDI*, *local government representative)*

#### Strategies to strengthening food labeling (S8 Table)

To increase the effectiveness of better food labeling, participants mentioned that providing public health education including how and why to read food labels is an important strategy to improve public awareness and application. Other strategies highlighted by participants included: mandatory labeling of nutrition declaration, which should be done by dialoguing with food companies and giving them enough time to adjust to the policy change on food labeling; creating standards for and enforcing front-of-package food labeling; simplification of labeling; and public education on the amount of salt in ultra-processed foods.

## Discussion

By interviewing a range of key stakeholders in the Federal Capital Territory (FCT), Ogun, and Kano states and using implementation science frameworks, this study provides insights on salt-related knowledge, attitudes, and behavior among stakeholders and explores their perspectives on the potential implementation outcomes, barriers, facilitators, and strategies for implementation and scale-up of Nigeria NMSAP priority actions to reduce excess dietary salt intake. While implementation of the four NMSAP priority actions and the strengthening of food labeling will be important to reduce excess salt consumption in Nigeria, there are multifaceted barriers that may affect implementation within and across these priority actions, but which can be addressed through cross-cutting and targeted strategies.

The most prominent barriers affecting two or more implementation outcomes included: consumer level barriers (poor knowledge of excess salt intake and its impact on health, distrust in government-led work due to poor implementation of existing dietary policies, cultural practices on food preparation), organization barriers (poor design of nutrition labels, perceived loss of customers due to changing taste), and policy level determinants (lack of salt replacement, poor advertising regulation, high cost of public health campaigns, existence of multiple sources of salt, bureaucratic nature of public institutions, affordability of high-salt foods).These implementation barriers are similar to those identified in previous studies of national salt reduction programs [26–29].

Participants noted that one cross-cutting facilitator of the four NMSAP priority actions and food labeling was the high media presence in Nigeria. Many participants in this study emphasized the important role of media in the implementation and scale-up of the Nigeria’s national salt reduction program. Since there is high media presence in Nigeria, this strategy provides a potential opportunity to use this avenue to widely educate the public on excess salt consumption. The starting point may be involving reporters in salt reduction program to increase media coverage to increase public knowledge and healthy behaviors related to salt and health [30]. Evidence showed that although use of media is an important strategy to implement the NMSAP priority actions including food labeling, it may be insufficient to create change in the absence of other strategies to create an enabling environment based on increases in knowledge, but not necessarily behaviors, through mass media campaigns [31]. Other important facilitators noted by participants for the implementation of the NSMAP priority actions include favorable existing advertising strategies on smoking, public interest in health issues, and existence of school feeding programs.

Key strategies suggested by participants in implementing the NMSAP priority actions include strengthening consumer education through multiple sources, front-of-package labeling, legislative initiatives to establish maximum salt content limits in foods and regular monitoring for implementation, mandatory front-of-package labeling schemes and warning labels for high salt foods for publicly procured foods and meals, strengthening regulations and implementation of food advertising, and interventions in public institution settings. Similar multifaceted national strategies to salt reduction have been reported in other countries [16,17,27,32]. For 96 countries that have implemented national salt reduction initiatives globally, three reported a substantial decrease (>2 g/day) in mean salt intake over time, nine reported a moderate decrease (1–2 g/day), and five reported a slight decrease (<1 g/day) over time [16].

Consumer education has been an important strategy for salt reduction globally, which has been mostly led by governments, followed by both government and NGO and food industry, and solely by NGO [15]. Participants in the present study stated that consumer education on salt reduction in Nigeria can be done through multiple sources such as religious and ethnic leaders, health professionals, and influencers and using local languages and dialects to minimize linguistic barriers to support grassroot mobilization. However, previous studies have shown that consumer education is more effective if complemented with fiscal incentives, such as government subsidies that lower the higher price of reduced-sodium salt [26,32] and structural or environmental interventions [33]. Hence, the Nigerian government should complement consumer education with fiscal and other policies to enhance impact of the NMSAP priority actions including food labeling. Previous evidence has demonstrated the positive impact of sugar-sweetened tax on reduction in sales and purchases of taxed beverages in the taxing countries. For instance, observed reductions in sales of taxed sugar-sweetened beverages after one year range from about 4% in Barbados [34] to 39% in a city in the US (Philadelphia) [35] and 58% for energy drinks in Saudi Arabia [36,37]. Implementing similar fiscal policies such as taxation of foods with high sodium content and subsidies for healthy foods may also help to reduce consumption of high-sodium foods in Nigeria [38]. Furthermore, participants suggested the need to enlighten consumers on how to quantify daily salt intake. However, this may be hard to do considering the complexities involved in quantifying daily salt consumption especially in commercially prepared foods [39]. Rather, it may be better to create a healthy food environment that help consumers lower their salt intake [40].

Participants in this study mentioned front-of-package labeling as an important strategy to reduce excess salt consumption in Nigeria. Evidence shows that front-of-package labeling is effective in improving public understanding and use of food labels and also support informed choices [41–43]. Front-of-package labeling has the potential to incentivize food manufacturers to reformulate their products to help consumers make healthier food choices by being able to identify foods with excessive amounts of specific nutrients including salts [41,44]. More than 30 governments worldwide have now implemented a front-of-package labeling system and at least 10 countries have made such labeling mandatory [44]. Similar to what the respondents in this study suggested, previous research showed that mandatory front-of-package labeling has been effective towards sodium reduction [44]. However, a voluntary approach appears to have limited public health impact [43,44]. The Nigerian government can learn from countries that have implemented mandatory front-of-package labeling to develop similar intervention towards national salt reduction in Nigeria. The current study’s data, along with previous reports, indicate that mandatory front-of-package labeling, which is under draft review in Nigeria in 2022, would be most effective.

Key informants also highlighted the importance of engaging the food industry and other stakeholders including consumers in the design of policy implementation work to reduce dietary salt including the priority actions and improving food labeling. This recommendation reflects other studies recommending a collaborative approach involving stakeholders across government, food industry, and individuals provides an important opportunity for implementing reduced sodium salt interventions [26]. This collaborative approach is important because while government may need to set salt reduction targets for food manufacturers, food industry needs to supply the reduced-sodium salt for food preparation to consumers, and consumers need to find these products acceptable before they will use the products [26]. However, even though collaborative approach is important towards salt reduction, the final decision on implementation of a national salt reduction program should rest with the government. For instance, even though participants in this study mentioned that food industry should be involved in government plans to change advertising approaches on salt reduction, keeping the food industry outside of the final decision-making table is generally recommended given the inherent conflicts of interest at stake [44].

Participants in this study explained the need for government to strengthen and enforce existing food and dietary policies in Nigeria. Even though there are existing dietary policies in Nigeria [45–47], these policies have not been fully implemented due to lack of clear implementation strategies and accountability mechanisms [47]. To reduce excess salt intake in Nigeria, the government should develop clear implementation strategies and accountability mechanism.

Some participants in this study also mentioned that ultra-processed foods such as packaged noodles and bread are major staple foods that have contributed to excess salt consumption in Nigeria, and this aligns with what previous studies have shown [48,49]. As a result, participants believed that reducing salt in these foods (i.e., packaged noodles and bread) may decrease consumption and possibly increase low-profit returns for food manufacturers. Nevertheless, previous studies in the Netherlands, Malaysia, Germany, and the United States have shown that using low-sodium salts in bread and noodles is feasible and widely acceptable to consumers without compromising taste or sales [50–53]. One additional challenge is the need to come up with strategies on how to decrease salt consumption without overly compromising the profit return of food manufacturers through increased cost or reduced consumption; doing this will increase adoption of the NMSAP priority actions among the food manufacturers in Nigeria.

Participants were very enthusiastic about the policy action of integrating nutrition education into school curriculum as a key strategy to long-term children’s behaviors towards excess salt intake. However, while nutrition knowledge may help students form positive behavior intentions towards healthy eating [54], there is no evidence that nutrition education is sufficient to change sustained actual behavior towards healthy eating, including among adults who are the target for achieving the Sustainable Development Goal Target 3.4 of reducing the risk of premature mortality from NCDs by 1/3 by 2030 [55].

Although this study engaged many stakeholders from government to non-governmental agencies, it has some limitations. First, our study was based on a purposive sample only including three states in Nigeria, and most came from FCT indicating that the results cannot be generalized to the whole of Nigeria. Similarly, the sample was not a representation of Nigerian population as participants had high baseline knowledge about salt intake and its impact on health. For instance, parents were not interviewed in this study. However, work is ongoing to ensure broad stakeholder engagement on this topic, including a November 2021 meeting at the authors’ institute that included >400 participants [20], as well as ongoing technical collaboration with the government to capture the quality of labeling and actual salt intake and knowledge at the population level. Further, this study did not achieve data saturation during this initial wave of interviews, but the study team will conduct additional interviews in subsequent waves in 2022 and 2024. Nevertheless, the stakeholders interviewed provided a wide range of opinions and experience on the potential barriers, facilitators, and strategies for effective implementation and scale-up of the four NMSAP priority actions on salt reduction including food labeling. Data on social marketing of salt consumption were not collected in the current study. These data may provide additional understanding on participants’ knowledge, attitudes and behaviors towards salt reduction, but this was beyond the scope of the current study.

## Conclusion

This qualitative study provides stakeholder perspectives on implementation and scale-up of the NMSAP efforts to reduce excess dietary sodium consumption in Nigeria. Many cross-cutting barriers and implementation strategies were identified to increase adoption, acceptability, and ultimately effectiveness of the NMSAP priority actions. While some of the participants’ narratives built on existing facilitators, many were targeting known or potential challenges, which will be important to address for the bold and important NMSAP to achieve its goal to reduce the risk of premature mortality from NCDs, including CVD, and to improve the health for all Nigerians.

## Supporting information

S1 Table Sample interview guide. (DOCX)

S2 Table Research checklist: COREQ (COnsolidated criteria for REporting Qualitative research) checklist. (DOCX)

S3 Table Contextual factors and implementation strategies for NMSAP priority action 1. (DOCX)

S4 Table Contextual factors and implementation strategies for NMSAP priority action 2. (DOCX)

S5 Table Contextual factors and implementation strategies for NMSAP priority action 3. (DOCX)

S6 Table Contextual factors and implementation strategies for NMSAP priority action 4. (DOCX)

S7 Table Barriers, facilitators and potential affected implementation outcomes for food labeling. (DOCX)

S8 Table Contextual factors and implementation strategies for food labeling. (DOCX)
