# Supplementary material for: Stakeholder perspectives on Nigeria’s national sodium reduction program: Lessons for implementation and scale-up
Source: PLoS One. 2023 Jan 13;18(1):e0280226. doi: 10.1371/journal.pone.0280226 (PMC9838847; doi:10.1371/journal.pone.0280226)
Supplement: S1 Table — (DOCX) [file pone.0280226.s001.docx]

**S1 Table. Sample interview guide.**

| 1. **How big of a problem do you think unhealthy diets are in your community in Nigeria in the development of chronic diseases like heart attacks, stroke, kidney disease, and high blood pressure? (outer setting, intervention [relative advantage])**    1. *Prompt: What do you think are the most important parts of a typical Nigerian’s diet to maintain or improve his or her health?*    2. *Prompt: What parts do you think will be hardest to change? How do you think that might differ, if at all, in different part of the country?*    3. *Prompt: How much of a problem do you think excessive salt in diets in Nigeria?*        1. *Probe: Why or why not?*       2. *Probe: Have you had any information around reducing salt in diet?*       3. *Probe: Where do you think Nigerians get the most salt in their diet?*          1. *Snacks, eating out, adding during cooking, other foods (e.g. bread)* 2. **Do you think most Nigerians in your community frequently look out at the contents of food they buy? Do you? (intervention [readiness])** 3. **If yes, then how do most Nigerians in your community look at the contents of food they buy? (intervention [readiness])**    1. *Prompt: Do most Nigerians read food labels?*       1. *Probe: Why do they read them?*       2. *Probe: Do they generally understand sodium or salt content?*       3. *Probe: Do people generally trust the information on the labels?*   ***Explain the key activities in the National Multisectoral Action Plan***  The government of Nigeria is working to reduce salt in food and diets to help decrease the burden of high blood pressure (hypertension) and other common, chronic diseases in Nigeria, like heart attacks, strokes and kidney disease. They have developed the Nigerian National Multisectoral Action Plan for the Prevention and Control of Noncommunicable Diseases. This includes a number of important actions to reduce salt in the foods people eat:   1. Limiting the amount of salt in in prepared foods or ingredients that you might buy. 2. Changing how companies can advertise their food, especially to children, to help improve healthy diets. 3. Public health campaigns to change how people learn about food, including limiting marketing of unhealthy food and beverages to children. 4. Education on nutrition in schools to make sure our children understand how to have a healthy diet.   **4. Tell me what you have heard, if anything, about the Multisectoral National Action Plan for the Prevention and Control of Noncommunicable Diseases so far. (outer setting)**   1. *Prompt: How did you learn about this? Where did you learn about this (or from whom)? (actors, inner setting)* 2. *Prompt: Are there previous examples of governmental programs that have been effectively implemented and scaled up in your community that might serve as a guide? (process of implementation)*   I would like to ask you about each of these 4 policy actions or interventions to understand your thoughts and what you think might work or be hard to accomplish, and what would be the best ways to help effectively implement and scale-up these policies.  **5. Policy intervention 1: Limiting the amount of salt in in prepared foods whether in ingredients or foods that you might buy (e.g. Maggi, dried fish, snacks) or if you eat outside the home. (intervention)**   1. *Prompt: What is your opinion about this approach in reducing salt and improving health in your community (attitude, appropriateness)?* 2. *Probe: Do you think it would be effective in reducing how much salt people eat?* 3. *Prompt: What are the things which would make this acceptable to you and most Nigerians in your community? (acceptability*) 4. *Probe: Cost? Taste?* 5. *Prompt: What are the things which would make this NOT acceptable to you and most Nigerians in your community? (acceptability*) 6. *Prompt: Who would be the people that need to be most convinced that this is important? (outer setting [peer pressure] and process of implementation [champions or external change agents])* 7. *Probes: Women? Working people? Leaders in the community? Healthcare workers?* 8. *Probe: How might acceptability differ for when limiting salt in cooking at home versus eating outside the home?*   **6. Policy intervention 2: Changing how companies can advertise their food, especially to children, to help improve healthy diets. (intervention)**   - 1. *Prompt: What is your opinion about this approach in reducing salt and improving health in your community? (attitude, appropriateness)*      1. *Probe: Do you think it would be effective in reducing how much salt people eat in your community?*   2. *Prompt: What are the things which would make this acceptable to you and most Nigerians in your community? (acceptability*)   3. *Prompt: What are the things which would make this NOT acceptable to you and most Nigerians in your community? (acceptability*)   4. *Prompt: What do you think needs to change in marketing to help people buy foods and eat less salty food in your community? (intervention [relative advantage])*      1. *Probe: Can you share an example of food marketing that promotes healthy eating with less salt?*      2. *Probe: What messages do you think would work in your community? (outer setting)*   **7. Policy intervention 3: Public health campaigns to educate people about health foods, including those low in salt, to help people in your community understand what steps to take for healthier eating. (intervention)**   1. *Prompt: What is your opinion about this approach in reducing salt and improving health in your community? (attitude, appropriateness)* 2. *Probe: Do you think it would be effective in reducing how much salt people eat in your community?* 3. *Prompt: What are the things which would make this acceptable to you and most Nigerians in your community? (acceptability*) 4. *Prompt: What are the things which would make this NOT acceptable to you and most Nigerians in your community? (acceptability*) 5. *Prompt: What messages do you think would be most helpful to people to learn about how to choose healthier foods to buy in your community? (intervention [relative advantage])* 6. *Prompt: How should these messages be delivered in your community (implementation)?* 7. *Probe: Radio, billboards, community organizations, social media, TV* 8. *Prompt: Who should deliver these messages, and why (or why not)? (implementation [external change agents], intervention, outer setting [needs])* 9. *Ministry of health or other government agencies* 10. *Health care workers* 11. *Community organizations such as nongovernmental organizations* 12. *Private industry* 13. *Other opinion leaders, including pop stars, religious leaders*   **8. Policy intervention 4: School-based nutrition education to make sure our children understand how to eat a healthy diet. (intervention)**   - 1. *Prompt: Do you have children? Are they school-aged? (outer setting [individual factor])*   2. *Prompt: What is your opinion about this approach in reducing salt and improving health in your community? (attitude, appropriateness)*      1. *Probe: Do you think it would be effective in reducing how much salt families in your community eat?*   3. *Prompt: What are the things which would make this acceptable to you and most Nigerians in your community? (acceptability*)   4. *Prompt: What are the things which would make this NOT acceptable to you and most Nigerians in your community? (acceptability*)   5. *Prompt: What messages do you think would be most helpful for school children in your community to learn about how to eat healthier foods? (intervention [relative advantage])*   **9. Do you know when implementation of any of these changes might begin in your community? (process of implementation)**   1. *Prompt: What would be needed to prepare for implementation of these policies in your community? (process of implementation)*   Thank you very much for your time and for answering these questions. Before we wrap up, is there anything else that you like to share? |
| --- |
