# Supplementary material for: Stakeholder perspectives on Nigeria’s national sodium reduction program: Lessons for implementation and scale-up
Source: PLoS One. 2023 Jan 13;18(1):e0280226. doi: 10.1371/journal.pone.0280226 (PMC9838847; doi:10.1371/journal.pone.0280226)
Supplement: S3 Table — (DOCX) [file pone.0280226.s003.docx]

**S3 Table.** **Contextual factors and implementation strategies for NMSAP priority action 1.**

| **Barriers (-)/ facilitators (+)** | **Implementation strategies** | |
| --- | --- | --- |
|  | **Strategies** | **Quotes** |
| Absence of nutrition facts label in processed foods (-)  Poor design of nutrition labels (-)  Limited understanding of information on nutrition labels (-) | Implement mandatory nutrition labeling (HP) | *In going about this salt reduction process, we can also go, we can also mandate all manufacturers the quantity of salt you used, should also be inscribed in the labels in a readable way that children, adult, all can read it. [IDI 012]*  *Well, if the policies on food labeling are very stringent and not allowing the companies to really take their time to change, that will force the companies to go ‘undercover’. What I mean by undercover is that they may produce something with salt and then they change the label. So, we must actually make the companies to understand these things, and once they understand it and they buy into it, if they buy into it, then they will be able to implement easily, but if they don’t buy into it they will actually cut corners and ensure that this policy doesn’t work. [IDI 008]* |
| Lack of knowledge on level and impact of excess salt intake (-) | Provide public education on the dangers of excessive salt intake (CL, FI, LSF, INGO, HP, FR, DT) | *I think the first approach is to let the public know the good and the bad sides of salt, since it cannot be totally eliminated from our foods. I think to get it done in a very short time; we start from the grassroots. [IDI 012].*  *Awareness creation, you know, using IC material, talk to people again; jingles; developing a radio magazine program, you know. Jingles. If you have people that…, most especially jingles, you know, a clip that will talk about the dangers of too much salt intake. Yeah. I think it will address to help to educate [IDI 004].* |
|  | Generate evidence on daily salt consumption (AC) | *Yeah, my opinion is that, yes, it’s a very good idea to do, but I feel that the first thing that we need to do is to establish the fact whether we (Nigerians) are actually are taking excess salt or not. Because that fact has not yet been established; because if we don’t know what is excess, we cannot start reducing. Because if we reduce it below what is normal, it also has its own detrimental effects. So, I feel that we should first of all find out what is the amount of salt. [IDI, academia]* |
| Misleading and deceptive labeling (-) | Improve knowledge of food manufacturers on the need for excess salt reduction (CL, FI, LSF, INGO, HP, FR, DT) | *I think there should be a proper enlightenment to these manufacturers, so they could understand the reason why this policy is about to be passed. Aside from enlightening manufacturers also, the government also need to enlighten the public on the need to reduce salt intake. [FGD 004]* |
| Lack of knowledge on and poor design of nutrition labeling | Improve nutritional labeling and product reformulation (HP, FI, LSF, AC, D, INGO) | *And then, there’s the one to also the general public, you know, maybe to tell them how they have to look out for labels – which one is healthy at what content is the salt healthy or not and all of that. [FGD 003]*  *In a way that manufacturer that is selling or advertising each of its products should look into the side of the children, maybe, by the way you write your food label, should also be readable by children as low as primary school. Let me deviate a little, in our contemporary time, I think the people that ask expired number than NAFDAC number in all our products now are the little ones. [IDI 012]* |
| - | Multi-sectoral collaboration and engagement | *But in a situation where there is no adequate collaboration between these stakeholders, a single organization cannot achieve it all… There should be this close collaboration between regulators and the industry; let’s start from the industry. We need close engagement with them, let them know the need, not just issuing decrees like the military regime. Let them know the importance of this regulation, why they need to reduce salt in manufactured products. [FGD 005]* |
| Concern about loss of customers because of changing taste | Develop policies that target the food industry to produce low salt products (CL) | *So, I think we should direct that policy mainly on those seasoning companies to control the content of salt they put in their seasonings. [IDI 004]*  *If the enforcement would be such that the quantity of salt, the level of salt that you should have in your food, I’m talking about manufacturers now, if they can tell manufacturers, this is the level, and they will enforce it and make sure that they stick to it. [IDI 018]* |
| Poor implementation of existing food and dietary policies | Develop strong government leadership, regulatory processes and accountability mechanisms (HP, FI) | *There’s some staples we adopted, somehow, somehow, they found their way into our own – bread and noodles. I think it’s something that wasn’t natural to us, which we have embraced, and it’s going to be an uphill task to tell people to not eat those things. So, what might now be the best is to regulate what goes into things like that. [FGD 001]*  *‘ think, beginning with the policy, it’s good, but the policy should have come when the law is in place. So, the opportunity is to revise the guideline and put a caveat for offenders. So, that when you come to implement the policy strategies, you have protection for the population.* |
| Evidence on daily salt consumption (+) | Conduct population survey to determine the actual amount of salt consumed in Nigeria (INGO) | *And then even working to limit the amount of salt in food, will also require some ground work like establishing baseline. What is even the present salt level content of the food. [FGD 003]* |
| Non availability of salt substitutes (-) | - | - |
| Mistrust in government due to poor implementation of existing food and dietary policies (-) | Using health care providers to inform patients and the general populace on the dangers of unhealthy, high-salted diets because people are likely to trust information provided by health care providers (+) | - |
| Lower cost of salt and (un)healthy diets (-) | - | - |
| Perceived long-term implementation of salt reduction program (-) | - | - |
| Cultural and social practices (-) | - | - |
| Existence of multiple sources of salt (-) | - | - |
| Non-availability of standard salt measurement in home-cooked meals (-) | - | - |
|  | - | - |

CL- Community leaders; FI- Food industry; LSF- Local, state and federal government; INGO- International NGOs; FR- Food retailers; HP- Health professionals; AC- Academia, RB- Regulator bodies, DT- Dietician
