# Supplementary material for: Stakeholder perspectives on Nigeria’s national sodium reduction program: Lessons for implementation and scale-up
Source: PLoS One. 2023 Jan 13;18(1):e0280226. doi: 10.1371/journal.pone.0280226 (PMC9838847; doi:10.1371/journal.pone.0280226)
Supplement: S4 Table — (DOCX) [file pone.0280226.s004.docx]

**S4 Table. Contextual factors and implementation strategies for NMSAP priority action 2.**

|  | **Implementation strategies** | | |
| --- | --- | --- | --- |
| **Barriers (-)/ facilitators (+)** | **Organizing theme** | **Basic theme** | **Quotes** |
|  |  |  |  |
| Poor advertising regulations (-) | Strengthen regulations and implementation on food advertising | Government to develop policy regulation on how food industry can advertise their foods. This can be done through increasing advertising costs, or develop a law against advertising of high-salt diets (LSF, INGO, CL, Dietician) | *A law to prosecute those who or companies that are manufacturing these things, that are not doing what the government already said, or group of people or professionals are doing, they should be prosecuted. [IDI 011]*  *In the issue of advertising their products, I think if they are having healthy stuff, it should be, maybe their cost – money for advertisement should be higher by so doing they will relax and then, maybe stop the production or they maintain the standards which the government wants. To comply, increase in cost, they will now feel like “no o, we’ll not be doing that again. (IDI 005)*  *For one, because you need to start with policy, go through legislation, and the legislators, and have the policy in place. Then, after, getting the legislation done and passed the bill on advertising, the other thing is focusing on how the advertising agencies will implement this policy that has been passed. So, I will say it’s a combination of policy and enforcement, because if you don’t enforce it, industries will not actually obey whatever is done (FGD 001)* |
|  |  | Advertising agencies to ensure that food industry conform to the laws and regulation that govern healthy diets (INGO) | *So, like the things that are happening around tobacco, are examples of what can happen. Then, after getting the legislation done and passed on advertising and other things is, the advertising agencies, how are they able to enforce the policy that has been passed. So, I will say it’s a combination of policy and enforcement, because if you don’t enforce it, industries will not actually obey whatever is done. (IDI 009)* |
|  |  | Front-of-pack nutrition labeling to enhance the effectiveness of advertising (HP) | *In marketing, I’ll believe that part of marketing is actually the branding, the outlook of the package –what they write on it, what is printed on it –you know, there are some products you go on the counter and see salted biscuits, salted this, salted that. So I believe that if there’s a way you can distinguish, okay this one has salt, this one has no salt, this one has low salt. So, maybe, once people know that I’m aiming for lower salt, people can have options of what to go for when they’re buying. [IDI 018]*  *(I)f such product will now have such bold label “low salt content” repeatedly, it will go a long way and make it acceptable by people. [IDI 010]* |
|  |  | Government to ban/sanction products that do not conform to the proper advertising regulation (PAG, Dietician) |  |
|  |  | There should be monitoring and supervision of all food advertisements (PAG) |  |
|  |  | Government to mandate the amount of salt to be used for processed foods. (LSF) | *We can also go, we can also mandate all manufacturers the quantity of salt you used, should also be inscribed in the labels in a readable way that children, adult, all can read it. [IDI 012]* |
|  |  | Food regulatory agencies to implement policies that ensure that all the food manufacturers declare the sodium content of food products on the labels (HP) | *So, all these noodles, it is important if the companies as they advertise probably put some, a very small piece of information to say, “This product is sodium regulated” or whatever caption they want to use. Just to show that, as long as this product is concerned, we are also concerned about your health, and therefore have ensured that the amount of sodium in this particular food product is within national acceptable limit for the country. (IDI 009)* |
| Favorable existing advertising strategy on smoking (+) | Leverage existing advertising approach on tobacco products | Similar tobacco advertising approach which prohibits advertising to children should be used for salt reduction (LSF, INGO) | *What tobacco has done is to enact a law that prohibits advertisement to children and anywhere around where children are prone to be available… I think the same can apply to food and beverage industries that are quite high or rich in salt and trans-fat, the same can apply to them as well. (FGD 005)* |
| Poor communication (by policy makers/experts) on the need to change how food companies advertise their food, especially to children, thus making it easy for this strategy to be hijacked by people with different vested interest in salt reduction (HP) | Strengthen the medium and nature of advertising on food | Use organizations drawn from the local community councils to enlighten the public before implementation of the policy will be beneficial (LSF) |  |
|  |  | Adverts and jingles on salt reduction should be done in local languages to enhance public understanding |  |
|  |  | Adverts should be educative and should inform the public about the danger of excess salt in advertised foods. | *So, if the advertisements can be more educative about the product than getting the consumers’ interest in consuming the goods. So, it should be rather more educative and telling them the risk of taking this one now. [FGD 004]*  *If you write that there's too much salt in it. Too much salt is not good for your health. [FGD 004]* |
| Possible resistance from the food industry due to the perceived threat on their sales (-) (LSF, INGO, HP) | Educate food industry on the need for salt reduction | Government needs to educate the food manufacturers to ensure that they (manufacturers) understand the gangers of excessive salt in their products (HP, INGO) | *Like I said they want to make profit and put few things, you know we like tasty things. So, it’s still back again to them, at least, awareness campaign that little of this, it is all of us, even them too, they will partake of it.* |
|  | Increase government support for food industry | Government to provide appropriate support to the food industry on how they can change the amount of salt in their products (INGO) |  |
|  |  | Government to provide incentives to food industry by giving subsidies in government media houses (INGO) |  |
|  | Multi-sectoral collaborations/stakeholder involvement | There is a need to involve the food industry in any plan to change advertising approach on salt reduction (FR) | *(I)dentify who those key stakeholders are, engage APCON, because I know they are the ones that set the standard and all of that, engage the other enforcement agencies…And then I'm sure we will be able to restrict the adverts of unhealthy food and unhealthy meals to the children. [FGD 003]*  *So, I think those are the issues that will make advertising to be well regulated. Of course, they will be push backs from the industry, but since health comes first, if the country is serious health will always prevail. [FGD 005]*  *We really need to sit down with professionals and then be able to sieve out, what do you actually call “healthy adverts”, as it concerns nutrition, you know; where you bring out professionals together, and they will brainstorm. [IDI 004]* |
|  | Increase government support for rebranding food industry advertising approach | Support food companies on how they can change their advertisement strategies qnd giving incentives such as subsidized advertising in government media houses. |  |
| High media presence in Nigeria/ collaborations with advertising companies (+) | Collaboration with advertising companies to promote the need for salt reduction. | Collaborate with advertising companies to promote the need for salt reduction will be an effective strategy towards salt reduction because of the wider reach of these advertising companies | *People look at this advertising companies so much – on news, radios, whatever; they give out handouts, they advertise with products, they give samples, you understand, free things, scholarships. So, they can make them friends, to do the right thing, and give them space to advertise and show what changed; the public will listen to them. (INGO)* |
| Consumer distrusts in brands and advertising (-) | - | - |  |

CL- Community leaders; FI- Food industry; LSF- Local, state and federal government; INGO- International NGOs; FR- Food retailers; HP- Health professionals; AC- Academia, RB- Regulator bodies, DT- Dietician
