# Supplementary material for: Stakeholder perspectives on Nigeria’s national sodium reduction program: Lessons for implementation and scale-up
Source: PLoS One. 2023 Jan 13;18(1):e0280226. doi: 10.1371/journal.pone.0280226 (PMC9838847; doi:10.1371/journal.pone.0280226)
Supplement: S5 Table — (DOCX) [file pone.0280226.s005.docx]

**S5 Table. Contextual factors and implementation strategies for NMSAP priority action 3.**

|  | **Implementation strategies** | | |
| --- | --- | --- | --- |
| **Barriers (-)/ facilitators (+)** | **Organizing theme** | **Basic theme** | **Quotes** |
|  | Platforms where public health campaigns should be carried out | Public health campaigns on salt reduction should be carried out in platforms where different audience could be reached: traditional media (TV, radio); social media; religious institutions (churches and mosques); public spaces (markets, motor parks) (CL, FI, INGO, HP) | *So many people like traditional rulers, from the mosque and churches will also play a vital role. But if you’re not a Muslim you will be either a Christian. So, once there is an information, what we do in our traditional way, when we talk to people, we invite the traditional community leaders and then the religious leaders as well. [IDI 015]*  *One of the places we need to do that enlightenment, is the market area. Because the population in the market area is big. Product enlightenment in a big market will also go a long way. [FGD 004]* |
| There are existing government institutions tasked with public health campaigns (+) | Multi-sectoral collaboration/involvement | There is a need for a multi-sectoral collaboration that brings different stakeholders together in promoting the public health campaigns on salt reduction. Such stakeholders should include:local, state and federal government; local and international NGOs; government agencies such as National Orientation Agency, Ministry of Women Affairs, Ministry of Education (LSF, FI) | *A lot of non-governmental organizations should also be involved. Media should be involved, to sensitize, to push, to ask questions. [IDI 004]* |
| Public interest in health issues (+) | Targets of public health campaigns | Public health campaigns on salt reduction should target different audience including children (because it is easier to change their consumption behaviors), parents (because they decide what the family buys and consume), religious leaders, traditional rulers, and ethnic leaders (CL, FI, INGO, HP) | *You catch them young.  When somebody is already an adult, he is already used to a particular eating behavior and will be hard to change. So, I think it will be helpful to center the campaigns around children. [IDI 021]*  *You know if you are starting something, there is what we call chances that you can give to encourage people to come, you can bring sweets or balloons, to bring to the children and then you can be teaching them. Even in churches, I even forgot to mention it, go to Sunday school, they should include it. Tell their imams, tell the pastors, through them, you’ll be able to penetrate. Most especially the Muslims, me I know they believe in their imams. So, by the time they know, penetrate those people, definitely, all the things will be accepted. So, it would help. [IDI 014]* |
| High level of illiteracy in Nigeria (-) | Communication/dissemination strategies | Use local languages in public education to get maximum impact and reach (CL, HP) | *(W)hat I would just want to add to that is that when we are going, when we go below the industry, the stakeholders, the whatever, when we are getting to the lower cadre, we should try to speak to them in their own languages. Let it get to the languages that everybody understands. And then, the message will be passed across. [FGD 002].* |
| High cost of public health campaigns (-) |  | Use trusted sources such as religious leaders, ethnic leaders, community health workers, community mobilizers, health professionals, community mobilizers, community health volunteers, teachers, social networks (LSF, HP, CL) | *The stakeholders and then, maybe secondly people that work in hospital, teachers, and traditional leaders and then we have the community mobilizers. So, people tend to understand the language of their own people and in schools. Like in schools now, if they should start that process, you’re growing up, they are teaching. Now if you ask a child now coming back from school, if you are trying to correct them, they will tell you “No, my teacher said this”. So, they tend to listen to their teachers. So, they’ll say “mommy they told us eating this salt is not good” [IDI 014]*  *People are also curious and worried about their health, and people listen to our clinicians a lot. So, if we have these people, if we have adverts and these people do advocacy and speak to people, people will be definitely listen; there will a lot of improvement with an attitudinal change with respect to salt reduction*. [IDI 009] |
| Poor internet services (-)  Favorable existing campaign on polio vaccination (+) |  | Grassroot mobilization to change how people learn about food (INGO, FI) | *I also think that for some other programs, what has worked is taking the message down to the grassroot, specifically for public health. So, for every local government in Nigeria, there are people they call, health educators or social mobilizers. And if we are able to pass this message along down through those educators that are part of these communities, I think we’ll go a long way. [FGD 001]*  *And also, being able to simplify the message in a way it’s easy to understand, so that even a town crier can pass the message. We can take examples from immunization and other things that they use even town criers to pass the message on what they need. Personally, I’ll start from the grassroot. [FGD 001]* |
|  |  | Use influencers such as music and movie stars, celebrities, and politicians to champion salt reduction public health campaigns (INGO) | *I can remember an example where the former president, Olusegun Obasanjo, one of the diabetic days, did a march with them, and told them you can live long with diabetes, that he is diabetic... because he is a diabetic, so you can see that he is a living example. So, those are the kind of champions we need even for salt intake, or salt reduction.*  *A lot of this generation, they like to copy stars. So, it’s very effective. (FGD 004)*  *Yes. Like we always do in Public Health, advocacy to the government; we can make one or two of them or a notable person within the government, we can make them champions of this campaigns. You can decide to make the Government, the Deputy Government or the commission a champion of salt reduction, so that when people like this come out publicly to say that you need to reduce your salt intake, the message can resonate. (IDI 002*) |
|  |  | Support public health campaigns with empirical data | *Because in convincing anybody, you have to show that person why, how, and so, when information is not available and facts are given and the link between these diseases and too much salt can be shown, I think people would, I think high percentage would listen. [IDI 018]* |
| Cultural background (-) | Message framing | Frame public health campaign messages in such a way that it shows the impact of excess salt intake on health challenges such as high blood pressure, cardiovascular disease and heart attacks. (FI, INGO, FR, HP) | *Yeah, if we, if we have good campaign and then they tell you the detrimental effect of this, it will make it to be acceptable. Because nobody wants to have any dangerous habit that will destroy him. So, by the time the campaigns actually show you this is what you have, and this is what is can give to you, that will actually make it acceptable to most of us. (IDI 008)* |
|  |  | Public health campaigns on salt reduction should focus on teaching consumers on how to quantify daily salt recommendation (HP) | *I think as earlier mentioned, the issue is that people don’t know this quantity that we call milligram or gram in our area, so if we can quantify the quantity of salt that should be enough, that we should not exceed that daily, then possibly divide it into three portions, so that everybody will know that in my breakfast, lunch or dinner, my salt intake should not exceed this quantity. It will go a long way. (IDI 010)* |

CL- Community leaders; FI- Food industry; LSF- Local, state and federal government; INGO- International NGOs; FR- Food retailers; HP- Health professionals; AC- Academia, RB- Regulator bodies
