# Supplementary material for: Stakeholder perspectives on Nigeria’s national sodium reduction program: Lessons for implementation and scale-up
Source: PLoS One. 2023 Jan 13;18(1):e0280226. doi: 10.1371/journal.pone.0280226 (PMC9838847; doi:10.1371/journal.pone.0280226)
Supplement: S6 Table — (DOCX) [file pone.0280226.s006.docx]

**S6 Table. Contextual factors and implementation strategies for NMSAP priority action 4.**

|  | **Implementation strategies** | | |
| --- | --- | --- | --- |
| **Barriers (-)/ facilitators (+)** | **Organizing theme** | **Basic theme** | **Quotes** |
| Children have a lot of influence on their families (+) | Integrate nutrition education into school curriculum | Integrate salt reduction and nutrition education into the school curriculum. Integrating nutrition education into school curriculum will bring about a sustained and prolonged influence on children and will ensure that low sodium intake is maintained into adulthood. In addition, this integration will bring about a long-term impact because children often trust their teachers and believe in what they are taught in school and can influence the consumption pattern of their families (LSF, INGO, AC, FI). | *It can also build into our curriculum, so that as the children is growing, the child should be informed, the salt, at least the harmful effects of salt. If it is taught in the school, I think it will go a long way. They are being indoctrinated to it, they are well equipped, and they can take the message even to their peers that are out of school. [IDI 012]*  *Because if the curriculum is reviewed to include some of these (nutrition education), it will be very helpful, as children will grow up with healthier behaviors and then, progressively leads to less diseases as they grow up. (IDI 018)*  *I told you that children tend to listen to their teachers even more than their parents. So, by the time that approach starts from the school, there’s no reason why it won’t stick to their heads. They’ll come back home and be telling their parents, “My teacher said we shouldn’t do this, my teacher said we should do that.” (IDI 014)* |
|  |  | Make information on nutrition education in the school curriculum simple and easy to understand by children (HP). | *As simple as possible. When I said it should be as simple as possible, there was a time when we were growing up and they were talking about too much carbohydrate and kwashiorkor, they made it, they gave us pictures, you saw it, and you will be afraid to be like that. So, when your parents tell you don’t eat rice, eat this, you’ll remember, and it drives home the point. So, I think something like that, if it is done, it will work for children. [IDI 018]* |
| Bureaucracy by school authorities (-) | Stakeholder involvement | Work with teachers and the entire school administrators to implement the salt reduction program (FI) |  |
|  |  | Engage the Ministry of Education for institutionalization of school-based activities and policies on salt reduction. (LSF) |  |
| Poor knowledge on appropriate salt intake levels among school staff (-) | Develop school-based policies on healthy diets | Develop school-based policies on healthy diets alongside education-based programs (LSF) |  |
|  |  | Chefs in boarding schools should be trained in proper nutrition and the need to prepare low-salt healthy diets (LSF) |  |
|  |  | Train the teachers to be able to teach healthy diets to children (AC, INGO) |  |
| Existence of school feeding program and school health programs in some schools (+) | Leverage existing programs and initiatives | Leverage on existing school feeding program by ensuring that low-sodium diets are provided to children (INGO) |  |
|  |  | Learn from successful initiatives that have led to change in children consumption behavior (AC) |  |
| - | Provide information to children on healthy eating and negative impact of excess sodium intake | Teach children on the benefits of healthy eating and impact of excess sodium intake using social media, poems, debate competition, cartoons, jingles, and use of ambassadors and celebrities and during classroom (LSF, HP, AC) | *Okay, to some extent yes, because, you know children whatever you tell them, they go back to the house, we were told that we should put small salt the food. So, it goes back again to the family, because at the end of the day, some of them are the ones that prepare this food. So, it’s already inculcated in their head that, “Okay we were told in the school, so let’s practice it”; and as they practice it, other members of the family will benefit from it.*  *You see most of the times, from the classroom to social media, because a lot of the children, these young ones prefer the social media. Anything they see in the social media they tend to accept it and think it is the right thing. So, for me, more of this talking should still be in the class and still be in the social media telling them that, a healthy diet, this is what it entails for you to be healthy, the message should have more of instructional materials that you would show to them clearly the effect of much salt in the body so that they can now shy away from it. [IDI 016]* |
|  |  | Practical demonstrations of healthy and low sodium foods in schools (Dietician) | *Let them also have a demonstration where the teacher can also do some kind of food demonstration, they will do well there. (IDI 011]*  *Teachers can be coming with food samples in demonstrating it to the children, they will learn. [IDI 011]* |

CL- Community leaders; FI- Food industry; LSF- Local, state and federal government; INGO- International NGOs; FR- Food retailers; HP- Health professionals; AC- Academia, RB- Regulator bodies, DT- Dietician
