# Supplementary material for: Stakeholder perspectives on Nigeria’s national sodium reduction program: Lessons for implementation and scale-up
Source: PLoS One. 2023 Jan 13;18(1):e0280226. doi: 10.1371/journal.pone.0280226 (PMC9838847; doi:10.1371/journal.pone.0280226)
Supplement: S7 Table — (DOCX) [file pone.0280226.s007.docx]

**S7 Table. Barriers, facilitators and potential affected implementation outcomes for food labeling.**

| **Organizing Theme** | **Barriers (-)/ facilitators (+)** | | **Implementation outcomes and effectiveness** |
| --- | --- | --- | --- |
|  | **Basic Theme** | **Quotes** |  |
| Poor labeling format/design | Absence of nutrition facts label in processed foods (HP, FI) | *A large number of these food are not even labeled neither are the contents or ingredients outlined. For example, when somebody takes eggroll, meat pie and things like that, the content and component are not mentioned. We just eat virtually what we find on the street so it’s a problem. [IDI 010]* | Adoption  Effectiveness |
|  | Tiny lettering of nutrition facts labels making it difficult to read (INGO, HP) | *Nigerians hardly look at food labels but rather depends on what they hear verbally. The labels also have tiny readings therefore discouraging readers. [IDI 010]*  *For one, the labels are very small, they take a very small proportion of those packs. So, most times, it’s even difficult to see what is written there. [FGD 001]* |  |
|  | Misleading and deceptive labeling (FI) | *They (consumers) just feel that these people just try to sell their market by putting in what suit them. For example we buy bread and you see a lot of bread contains a label “no saccharin or no sugar added or no saccharin added or no bromate added”; but then, that particular food contains bromate. [IDI 008]* |  |
|  | Difficulty in understanding nutrition labeling due to use of technical terms (FI) | *-* |  |
| Distrust in labels | Lack of trust in food labels because labels may not be a true reflection of the food content (FI, LSF, AC, D, INGO) | *Yes, but not all labels. Most especially the locally made because they feel from experience like some people have said, they feel that sometimes even if the thing expire, they remove the label and put a new one. So, some people don’t trust it. [IDI 014]*  *Although people trust some labels. However, people do not trust all the food labels, especially the locally made products, because they think that sometimes even if the product expires, they (food manufacturers) can remove the label and put a new one. So, some people don’t trust it. [IDI 005]* | Acceptability  Effectiveness  Fidelity |
| Lack of knowledge of label use/value | Lack of knowledge on appropriate salt levels (CL, FI, LSF) | *(B)ut a normal person who is healthy, I’m not sure they look at salt or even if they do, I’m not sure they even know what quantity they should be looking out for, that okay, this is the minimum I should get or maybe there’s a way to calculate the amount of salt I’ve taken today. [IDI 018]*  *They look at all the things that are there but most of them don’t even look at it and see whether they have large amount of salt in the diet or not”. What most Nigerians check is the expiry dates. How many Nigerians even know what the normal amount of salt is? [IDI 014]* | Acceptability  Appropriateness |
|  | Lack of knowledge on the benefits/value of reading food labels (INGO, FR, AC, LSF). | *You know, it’s awareness that drives behavior and attitude. So, because people are not even aware of the benefits of checking food labels in the first place, they don’t likely check for the content of sodium in food or food products. [FGD 001]* |  |
| Different goals of looking at labels | Checking food products for other things such as expiry dates, calories, sugar and NAFDAC code and not food labels for salt (CL, FI, LSF, INGO, HP, AC, RB). | *They look at all the things that are there but most of them don’t even look at it and see whether they have large amount of salt in the diet or not”. What most Nigerians check is the expiry dates. [IDI 014]*  *Majority of us don’t care (about checking food content for salt). Majority of individuals don’t take note of these things, they consume first. …and it’s after consumption before they look out for these things…same attitude of not reading medication labels. [IDI 019]* | Acceptability  Appropriateness  Feasibility |

CL- Community leaders; FI- Food industry; LSF- Local, state and federal government; INGO- International NGOs; FR- Food retailers; HP- Health professionals; AC- Academia, RB- Regulator bodies
