# Supplementary material for: Stakeholder perspectives on Nigeria’s national sodium reduction program: Lessons for implementation and scale-up
Source: PLoS One. 2023 Jan 13;18(1):e0280226. doi: 10.1371/journal.pone.0280226 (PMC9838847; doi:10.1371/journal.pone.0280226)
Supplement: S8 Table — (DOCX) [file pone.0280226.s008.docx]

**S8 Table. Contextual factors and implementation strategies for food labeling.**

|  | **Implementation strategies** | | |
| --- | --- | --- | --- |
| **Barriers (-)/ facilitators (+)** | **Organizing theme** | **Basic theme** | **Quotes** |
| Lack of knowledge of label use/value (-) | Increase public education on labeling | Public education on the importance of checking food labels for salt (CL, FI, LSF, INGO, HP, AC, RB). | *Going forward, people should be given education on the need to look out for nutritional fact table that shows sodium or salt content, and protein, carbohydrate, and things like that from food products purchased in shops. [FGD 002]* |
|  |  | Provide public education on how to read food labels (FI, LSF, AC, D, INGO) | *And then, there’s the one to also the general public, you know, maybe to tell them how they have to look out for labels – which one is healthy at what content is the salt healthy or not and all of that. [FGD 003]* |
|  |  | Public education highlighting the benefits of reading food labels (INGO, FR, AC, LSF). | *You are what you eat”, that is what they will say. And when they read food label showing that oh this particular product has this and they know they are not supposed to eat it, they will get away from it. [IDI 021]*  *Like I have said, I said, they should just maybe ban such products. And then, once people have, I’ll just keep on saying it, proper education on certain things, to be checking the labels, I said that earlier on, if they are checking the labels, they will know. [IDI 005]* |
| Absence of nutrition facts in processed foods (-)  Tiny lettering of nutrition fact labels (-) | Mandatory labeling | Mandatory labeling of nutrition declaration. This should be done by dialoguing with food companies and giving them enough time to adjust to the policy change on food labeling (HP). | *In going about this salt reduction process, we can also go, we can also mandate all manufacturers the quantity of salt you used, should also be inscribed in the labels in a readable way that children, adult, all can read it. [IDI 012]*  *Well, if the policies on food labeling are very stringent and not allowing the companies to really take their time to change, that will actually force the companies to go ‘undercover’. What I mean by undercover is that they may produce something with salt and then they change the label. So, we must actually make the companies to understand these things, and once they understand it and they buy into it, if they buy into it, then they will be able to implement easily, but if they don’t buy into it they will actually cut corners and ensure that this policy doesn’t work. [IDI 008]* |
| Misleading and deceptive labeling (-) | Front-of-pack nutrition labeling | Enforce front-of-pack nutrition labeling (HP) |  |
| Use of technical terms making labels complex to understand (-) | Label simplification | Simplification of labeling such that everyone can understand, including children (FI). | *In a way that manufacturer that is selling or advertising each of its products should look into the side of the children, maybe, by the way you write your food label, should also be readable by children as low as primary school. Let me deviate a little, in our contemporary time, I think the people that ask expired number than NAFDAC number in all our products now are the little ones. [IDI 012]*  *In going about this salt reduction process, we can also go, we can also mandate all manufacturers the quantity of salt you used, should also be inscribed in the labels in a readable way that children, adult, all can read it. [IDI 012]* |
| Lack of knowledge on appropriate salt levels (-) | Increase public education on appropriate level of salt | Public education on the appropriate amount/level of salt to look out for in processed foods (CL, FI, LSF). | *Well, just as I said, the few that check, some look at the salt content, and, but the other thing there is, I would say, it is even better that everybody knows the quantity of salt or the quantity of sodium chloride that is needed to be injected to the body per day. It could differ from adult, it could differ by age, it could also differ maybe let’s say you are at the age of two to three, what is the quantity of salt you need to take per day. Or if you already have certain ailments what is the quantity of salt you need to take per day or per meal or per week. [IDI 012]*  *You are what you eat”, that is what they will say. And when they read food label showing that oh this particular product has this and they know they are not supposed to eat it, they will get away from it. [IDI 002]* |

CL- Community leaders; FI- Food industry; LSF- Local, state and federal government; INGO- International NGOs; FR- Food retailers; HP- Health professionals; AC- Academia, RB- Regulator bodies
